# Backward-Looking Principles of Climate Justice: The Unjustified Move from the Polluter Pays Principle to the Beneficiary Pays Principle

**DOI:** 10.1007/s11158-022-09569-w

**Published:** 2022-11-08

**Authors:** Laura García-Portela

**Affiliations:** Environmental Sciences and Humanities Institute, University of Fribourg, 1700 Fribourg, Switzerland

**Keywords:** Climate justice, Polluter pays principle, Beneficiary pays principle, Causation, Excusable ignorance, Fairness, Legitimate expectations

## Abstract

Climate change involves changes in the climate system caused by polluting human activities and the social and natural effects of these changes. The historical and anthropogenic grounds of climate change play an important role in climate justice claims. Many climate justice scholars believe that principles of climate justice should account for the historical and anthropogenic sources of climate change. Two main backward-looking principles have been proposed: the polluter pays principle (PPP) and the beneficiary pays principle (BPP). The BPP emerged in the literature on climate justice in response to certain objections raised against the PPP. In this paper, I focus on two of these objections: the causation objection and the excusable ignorance objection. Defenders of the BPP have traditionally assumed that this principle is not vulnerable to those objections, which renders the BPP superior to the PPP. In this paper, I challenge this underlying assumption. My argument here is simple: moving from the PPP to the BPP in response to any of these objections might be unjustified because the BPP is affected by at least some of the considerations giving rise to these objections.

## Introduction

We know that climate change has been caused by emission-generating activities throughout history and very intensively since the period of industrialization (IPCC 2014). This fact has raised claims of historical responsibility for climate change both in the political debate and in the general civic debate (Friman and Hjerpe 2015). Famously, Barack Obama stated at the COP21 in Paris in 2015: ‘I’ve come here personally, as the leader of the world’s largest economy and *the second-largest emitter*, to say that the United States of America not only *recognizes our role in creating this problem*, we embrace our responsibility to do something about it’.^1^ Obama’s words suggest that how climate change came about might be a relevant consideration for the distribution of burdens in addressing climate change. Climate scholars have tried to make sense of this concern by invoking backward-looking principles of climate justice.

The most prominent backward-looking principles are the polluter pays principle (PPP) and the beneficiary pays principle (BPP).^2^ In a nutshell, the PPP states that polluters should bear the burdens associated with emission-generating activities because they are responsible for their adverse effects. This principle follows the maxim that if ‘you broke it, you fix it’ (Caney 2006, 2010) or ‘clean up your own mess’ (Shue 1999; Gardiner 2016). The BPP states that those benefitting from climate-change-inducing activities should bear the burdens associated with tackling the adverse effects of climate change. The BPP emerged in climate justice literature as an alternative to the PPP based on certain objections pressed against this first principle. Often, defenders of the BPP have claimed that this principle preserves the backward-looking elements of the PPP, but it solves the practical and theoretical challenges associated with the first principle. In this way, the BPP is taken to provide a better account of our backward-looking intuitions concerning climate justice.

Very often, defenders of the BPP assume that their principle is free from the problems associated with the PPP. In this paper, I focus on two objections: the causation objection (CO) and the excusable ignorance objection (EIO).^3^ Various scholars have argued that the BPP does not encounter at least one of these objections, which provides a reason to prefer the BPP over the PPP (Gosseries 2004; Meyer and Roser 2010; Caney 2010; Bell 2011; Page 2012; Meyer 2013; Baatz 2013; Duus-Otterström 2014; Heyd 2017). Here, I challenge that underlying assumption. I argue that shifting from the PPP to the BPP based on either of these objections might be unjustified because the BPP might be affected either by the same objections or by the same considerations that gave rise to the objections. I remain agnostic on what this means more generally for backward-looking principles.

I proceed as follows. In Sect. 2, I introduce the two objections. In Sect. 3, I present the BPP in this context and explain how it can be understood as evading these objections. In Sects. 4 and 5, I show how these two objections might affect the BPP. In Sect. 4, I argue that the CO affects both principles. In Sect. 5, I offer an interpretation of the EIO and the fairness considerations that might motivate this objection and argue that the BPP is also affected by those considerations. Finally, I conclude the paper by recapitulating my main argument.

One important caveat should be noted before starting. The results of this paper concern all climate change-related duties: mitigation, adaptation, and compensation. However, some of my arguments concern only *some* of these duties. For instance, my discussion of the CO focuses on the application of the PPP and BPP to the distribution of burdens when dealing with harm in specific geographical locations. Such harm might occur in the short- or mid-term, and thus calls for adaptation, or it may have already occurred, and thus calls for compensation. Therefore, my discussion here is limited to adaptation and compensation duties and does not concern the application of these principles to the distribution of mitigation duties. In contrast, my analysis and conclusions concerning the EIO apply to all climate change-related duties.

## Two Objections Against the Polluter Pays Principle: The Causation Objection and the Excusable Ignorance Objection

The CO states that without clear knowledge about which specific weather events are caused by emission-generating activities polluters cannot be made to pay for the negative effects associated with those weather events, or extreme weather events (EWEs) (Adler 2007; Caney 2010; Farber 2017; Wallimann-Helmer et al. 2018). The reason is that we simply might not know whether those EWEs have occurred or will occur due to polluting activities at all. Climate scientists are confident that climate change has been caused by greenhouse gas (GHG) emissions. They are also highly confident that slow-onset events such as sea-level rises are caused by climate change (IPCC 2014). However, it is more difficult to know whether particular EWEs (such as extreme temperatures, heatwaves, droughts, floods, extreme rainfall, etc.) have been or will be caused by anthropogenic influences on the climate system, because EWEs can also happen due to natural variability in a world without climate change. Although detection and attribution studies have improved significantly in the last decade (Stuart-Smith et al. 2021), they are still in development and not free of important objections and problems (Huggel et al. 2015; Shepherd 2014).

Note that this objection affects the application of the PPP to adaptation and compensation duties but not to mitigation duties. Mitigation duties might be required because we know that climate change will likely cause harm in the future and that additional emissions are likely to contribute to that harm, although we do not know exactly *where* that harm will occur.^4^ Nothing of that requires connecting emissions-generating activities with the local manifestations of climate change. But adaptation duties (understood as *climate change-related* duties) require knowing which regions are projected to be affected by (at least some) weather events that have climate change as their main driver. Thus, if adaptation duties ought to be distributed to polluters, a connection between emissions and the location of those foreseeable harmful impacts needs to be established. Otherwise, polluters could rightfully ask why their money is used to tackle foreseeable harm without knowing whether that potential harm is connected to their emissions. Likewise, those seeking compensation for the harmful impacts of climate change based on the PPP need to show a connection between polluters' emissions and the harm they suffered.^5^

The EIO states that no one should be held accountable for the effects of their actions if these were unknown and could not reasonably have been foreseen. Arguably, this is the case for historical emissions. Before the publication of the First IPCC Report in 1990, the scientific community had not yet reached consensus about the adverse effects of GHG emissions on the climate system. Governments and citizens alike could not be expected to know about the adverse effects of emission-generating activities. Many scholars have considered this objection to be one of the most powerful against the application of the PPP for pre-1990 emissions (Gosseries 2004; Caney 2006, 2010; Bell 2011; Page 2008, 2012; Meyer and Roser 2010; Wündisch 2017). This objection has also been relevant in climate negotiations (Gardiner 2016, p. 111). Admittedly, the objection is relatively limited because it only affects emissions emitted until roughly 1990. However, and importantly, these historical emissions still represent around half of total global emissions.^6^

Unless we want to challenge the empirical assumptions concerning the availability of information about the negative effects of climate change and the role of emission-generating activities, the only way to apply the PPP to historical emissions is as a principle of strict liability. That is, the PPP needs to be understood as a principle that allocates to an agent the duty to deal with the harm associated with her action, phi, ‘irrespective of any steps that she took in order not to phi and irrespective of whether she knew or had reason to know that she was phi-ing [including any steps she took to find out whether she was about to phi]’ (Gardner 2011, p. 207). This understanding of the principle departs from one that relies on attributions of culpability and that seeks to punish emitters, at least for historical emissions. In the following, for the sake of the argument, I propose to accept the empirical assumptions behind the EIO and, thus, to accept that the PPP can only work as a principle of strict liability when applied to historical emissions. Moreover, my discussion will be based on the objections that the PPP faces as a principle of strict liability.^7^

These objections have led some scholars to propose an alternative principle, the BPP, at least to cover duties related to a part of overall emissions. In the next section, I introduce this principle in more detail and show how, at first glance, it could presumably surmount these objections.

## The Beneficiary Pays Principle and Some Intuitive Reactions to the Objections

A more general version of the BPP states that beneficiaries of an injustice should bear the burdens associated with the injustice. With climate change, the principle is generally understood as stating that those benefitting from emission-generating activities should bear the burdens associated with tackling climate injustice or the harmful effects of climate change.^8^ In the context of climate change and historical emissions, the principle is most widely understood as possessing the three salient following characteristics, which define the version of the BPP that I discuss in this paper.

First, the BPP usually assumes that the beneficiaries are innocent. This means that in receiving their benefits they did not do anything for which they can be considered culpable, such as inducing or participating in unjust or potentially harmful actions. Consequently, it is not their culpability that grounds their duties. Second, the principle does not require that the original perpetrators (i.e. emitters) are culpable for their unjust or harmful actions. Thus, the principle applies when the emitters from whom beneficiaries receive their benefits did not know about the negative consequences of climate change or when they could have not avoided engaging in climate change-inducing activities. This characteristic allows the principle to account for historical emissions, and thus it supports the most overarching version of the BPP when applied to climate change.^9^

Third, the principle is usually understood to be victim-centred in two ways: (i) The principle is motivated by a concern for the actual or potential victims and it seeks to prevent or alleviate their suffering (Baatz 2013, 2016; Lawford-Smith 2014; Couto 2018). (ii) The principle is also victim-centred in a more specific way, because it is not only focused on solving the situation of victims of injustices or undeserved harm in general. It is also focused on the suffering of those victims that are likely to be affected by the activities from which the beneficiaries, and thus potential duty-bearers, benefit. This feature is grounded in the common source of benefits and disadvantages, which often operates as a justification of the principle (Duus-Otterström 2017). As Page has put it: ‘profiting from activities that impose climatic disadvantages … here, triggers a remedial duty on the part of the beneficiaries …solely because the disadvantages and benefits share *common origins* (Page 2012, p. 313).^10^ In the same vein, others have argued that this is a principle that seeks to even out the benefits and potential harm associated with climate change (Meyer and Roser 2010; Baatz 2013; Meyer 2013).

This victim-centredness characterizes the version of the BPP that I address here. Alternative versions remain excluded from my discussion. Nonetheless, I believe that this feature makes this principle a genuine principle of climate justice in comparison to other versions of the BPP.^11^ Take, for instance, a purely beneficiary-centred version of the BPP. The rationale of this principle is that it is wrong in itself for the beneficiary to keep certain benefits associated with injustices or undeserved harm because they are tainted, and the point of this principle is to require beneficiaries to just surrender their benefits (Couto 2018, p. 2172). Thusly defined, this principle can hardly work as a proper principle of climate justice because, arguably, a principle of climate justice requires not only removing resources from certain agents but also using them to solve certain problems caused by climate change. Or take, for instance, an ‘undirected disgorgement BPP’ (Duss-Otterström 2017; similar Goodin 2013). According to this principle, tainted benefits acquired from unjust or harmful activities should not only be given up, as with a purely beneficiary-centred principle, but should also be added to society’s general pool of resources and eventually used to solve injustices or alleviate undeserved harm. Nonetheless, this principle cannot work as a proper principle of climate justice when thus described because a principle of climate justice arguably requires certain resources to be used to address the actual or potential undeserved harm associated with climate change. Neither principle can properly work as a principle specifically of climate justice: a principle that seeks to tackle climate injustice, the harmful effects of climate change, and other problems associated with climate change.

The BPP has often been described as a hybrid principle that combines backward-looking aspects with forward-looking ones and that has the advantages of both without their disadvantages (Page 2012). Forward-looking principles consider the reasons we have today to make the world a better place now and usually take into account individuals’ abilities to bear these burdens. This BPP relies partly on a forward-looking rationale because it is focused on alleviating underserved harm and relies on agents’ ability to solve the problem with the benefits they received. However, it is also backward-looking because it does not ignore the sources of the problem and ‘isolates for redistribution only those benefits that are strongly connected to *climate change producing acts*’^12^ (Page 2012, p. 313).

Advocates of the BPP have proposed this principle as an alternative to the PPP. Many climate justice scholars have moved, in one way or another, from the PPP to the BPP in response to at least one of the objections introduced in the previous section (see Gosseries 2004; Meyer and Roser 2010; Caney 2010; Bell 2011; Page 2012; Meyer 2013; Baatz 2013, 2016; Duus-Otterström 2014; Heyd 2017).^13^ Interestingly, however, defenders of the BPP do not address how this principle can circumvent these objections. Instead, they only assume that it can do so.

Admittedly, there might be reasons to believe that the BPP is not open to these objections. First, concerning the CO, this principle does not focus on the link between emission-generating activities and their effects on certain geographical locations. Second, it seems to be unaffected by the EIO because the grounds for making beneficiaries bear the burdens associated with climate change are the resources that they might have enjoyed, not the actions that caused climate change. These reasons could explain the assumption that the BPP is not open to the objections pressed against the PPP. However, I believe that a closer examination shows that the BPP is indeed vulnerable at least to the concerns underlying these objections.

## The Causation Objection and the Beneficiary Pays Principle

To see whether the BPP also relies on the type of connection required by the CO, we need to recapitulate and explore the principle a little more. The principle requires beneficiaries of emission-generating activities to bear the burdens associated with addressing the adverse effects of climate change. The principle ‘isolates for redistribution only those benefits that are strongly connected to *climate change producing acts*’ (Page 2012, p. 313).^14^ That is, the benefits that are up for redistribution are those coming from emission-generating activities. This reference to the link between benefits and emission-generating activities appears repeatedly in the literature on the BPP. Similarly, Meyer (2013) has highlighted that ‘the goods in question [whose redistribution is called for] are the benefits that people realize in *carrying out actions that unavoidably have emissions as a side-product*’ (Meyer 2013, p. 600).^15^ The underlying assumption in the current discussion is that beneficiaries should bear the duties associated with tackling climate change because they have benefited from the activities causing the problem, which are emission-generating activities (see also Caney 2010; Baatz 2013; Schüssler 2011; Karnein 2017).

Conversely, we have the burdens that are to be paid for with these benefits. As we saw above, the relevant burdens are those associated with undertaking or paying for adaptation and compensation. Moreover, as we saw before, the ‘common source’ justification for the BPP excludes beneficiaries of emission-generating activities from the duty to bear burdens associated with injustices or undeserved harm unconnected to GHG emissions, such as burdens associated with addressing the negative effects of unjust wars, terrorist attacks, sexual assaults, gender violence, and rare diseases. Most importantly, they also exclude the injustices or undeserved harm caused by environmental impacts resulting from natural variability. None of this (potential) harm is derived from emission-generating activities, and therefore, it is not the duty of these beneficiaries to bear the burdens of preventing or alleviating it, according to this principle. Thus, attributing duties to alleviate climate change-related harm to beneficiaries of emission-generating activities requires discriminating between weather events caused by anthropogenic forcing and those caused by natural variability.

These remarks show that the BPP runs into the CO for both compensation and adaptation burdens. Let us start with compensation burdens, for which the application of the CO is perhaps clearer. According to the BPP, those benefitting from GHG emissions are the ones who should bear the burdens of compensation because those emissions both benefitted them and caused harm. The rationale behind the principle is that beneficiaries of emissions should respond to the harm caused by the emissions because benefits and harm share the same source: the emissions. But this does not seem to sidestep the problem of causation, because the GHG emissions would still need to be shown to be involved in the causation of the harm for which compensation is required. The connection between emissions and the harm still needs to be proven. If causation were not proven but we used the benefits of GHG emissions to compensate those affected by environmental harmful events happening in specific geographical locations, we would run the risk of using these benefits to address the harm that does not share a common source with the benefits. But this is not what the benefits are for, according to the BPP. Therefore, applying the BPP to compensatory duties requires the demonstration of a causal link between GHG emissions and their effects. *As long as* we think this is a difficult or even an impossible task, the BPP runs into the CO.

Is this any different for adaptation to the foreseeable harmful effects of anthropogenic climate change? I do not think so. According to the BPP, those benefitting from GHG emissions are the ones who should bear the burdens of adaptation because those emissions both benefitted them and will foreseeably cause harm to third parties if adaptation measures are not undertaken. The rationale behind the principle is that beneficiaries of emissions should avoid the potential harm that would be caused by emissions because their benefits and that potential harm share the same source: the emissions. Again, this does not seem to sidestep the problem of causation. The GHG emissions would still need to be shown to be significantly involved in the causation of the foreseeable harm for which adaptation is required. If causation were not proven but we used the benefits of GHG emissions for adaptation measures to avoid the foreseeable harmful events happening in specific geographical locations due to weather events, we would run the risk of using these benefits to address the potential harm that does not share a common source with the benefits. Remember: those weather events might still be caused by changing environmental conditions that are not due to climate change. But the BPP is not meant to distribute burdens for avoiding the foreseeable harmful impacts of weather events that are not due to climate change, as it is not meant to distribute burdens concerning the foreseeable harmful effects of rare diseases or terrorist attacks. Arguably, it would be unfair to ask beneficiaries of emissions to bear the burdens of adaptation to anthropogenic climate change if we do not even know whether those foreseeable harmful effects will be caused by anthropogenic climate change (i.e. emissions-generating activities) at all. Therefore, applying the BPP to adaptation duties requires the demonstration of a causal link between GHG emissions and potential harm. *As long as* we think this is a difficult or even an impossible task, the BPP runs into the CO also when applied to adaptation duties.

## The Excusable Ignorance Objection and Fairness Considerations

The EIO states that no one should be held accountable for the effects of their actions if these were unknown and could not reasonably have been foreseen. However, one might wonder why this is the case. Climate ethicists have rarely provided a deeper justification for the EIO: they rarely explain what the problem is with holding someone accountable for the effects of their actions when these were undertaken under circumstances of excusable ignorance. Instead, they merely state that excusable ignorance is a problem when trying to hold agents liable for historical emissions.

Various reasons might be advanced to reject holding people accountable for the effects of their actions if these were unknown and could not reasonably have been foreseen. However, an exhaustive analysis is beyond the scope of this paper. Instead, I would like to propose and explore one possible interpretation of the concerns motivating the EIO. I argue that the EIO might be motivated by fairness considerations and that these considerations apply not only to the PPP but also to the BPP. Hence, if I am right, this conclusion precludes moving from the PPP to the BPP in response to the EIO.^16^

### A Fresh Look at the Excusable Ignorance Objection

In this subsection, I offer a plausible interpretation of the motivation behind the EIO in two steps. First, I show that the concern behind the EIO might not be best expressed by saying that people should not be held accountable for the effects of their actions if these were unknown and could not reasonably have been foreseen. Instead, the concern might be that people should not be required to bear burdens they could not have expected being required to bear. I support my interpretation by showing how retrospective and prospective principles of strict liability are unequally affected by the original formulation of the EIO. Second, I argue that this more precise formulation of the EIO might be supported by fairness considerations: making people bear these burdens is unfair because it frustrates their legitimate expectations and truncates their ability to plan and execute their mid- and long-term life plans.

To begin, we first need to clarify the kind of principle against which the EIO is usually raised in climate justice debates. As we saw, this objection affects the PPP applied to pre-1990 emissions. The PPP works here as a principle of strict liability. Such a principle imposes liability for the harmful effects of actions ‘irrespective of any steps that she took in order not to phi and irrespective of whether she knew or had reason to know that she was phi-ing [including any steps she took to find out whether she was about to phi]’ (Gardner 2011, p. 207).

We should differentiate between prospective and retroactive strict liability.^17^ Prospective strict liability, or strict legal liability, is applied when principles of strict liability already exist as part of some regulatory scheme or legal system and their justification lies in the future-orientated implications of these principles. This understanding of strict liability is common in regulations concerning environmental pollution.^18^ For instance, a strict liability principle may state that if a chemical company pollutes a river, the company will be held liable for the environmental damage caused by the pollution regardless of whether the company made all reasonable efforts to avoid this damage and regardless of whether the company had good reasons to believe that these would be the results of its action. This feature makes this a principle of strict liability, but its justification is forward-looking. The existence and application of such a principle are justified by the general and prospective beneficial consequences of holding companies, people, and other entities liable for the harmful effects of their actions when they manipulate potentially harmful substances. In such circumstances, a principle of strict liability may be justified as a way of distributing the risks and costs of damages effectively. It may well be that making those who cause harm pay for the adverse effects of their actions is the best way to ensure that people take reasonable steps to avoid harm or even that it is the best way to ensure that costs do not fall on victims.

Note that this prospective justification requires that everyone must have the opportunity to be aware of it: information about the applicability of strict liability must be disseminated ahead of time in a way that any person can have reasonable access to it. Further, agents must have a choice about whether and how to participate in the activities regulated by these principles of strict liability (Wündisch 2017, p. 845). Arguably, if these principles are to disincentivize certain activities and make people maximally careful, they need to be widely known in advance. Thus, people are aware of the costs they might incur by engaging in certain activities and can decide whether and how to engage in these activities. Otherwise, the disincentivizing mechanism will not work.

Usually, proponents of the EIO do not object to this application of strict liability. They even agree that these principles are a good way of distributing the risks associated with certain potentially harmful substances. A system of strict liability, they acknowledge, may establish the right ‘incentives either to refrain from especially dangerous activity altogether or to be exceedingly careful when engaging in it’ (Moellendorf 2014; similarly Bell 2011 and Wündisch 2017). They believe that the EIO does not apply in these circumstances because, although those causing harm may not have been able to know about the negative effects of their actions, they were informed beforehand of the burdens they would need to bear in case of accidents, and yet they decided nonetheless to undertake these actions.

The situation is different for retrospective strict liability. Strict liability is applied retrospectively when no pre-existing legal scheme assigns liability for the negative effects of certain actions. Agents are held liable for the effects of their actions regardless of whether they made all reasonable efforts to avoid these effects and regardless of whether they had good reason to believe that the effects would result from their actions. However, unlike prospective strict liability, people are not informed beforehand of the burdens they need to bear for the possible negative effects of their actions. Instead, the causal connection between agents’ actions and their negative effects is taken to be morally relevant to attributing liability for the costs of the negative effects.

To summarize, prospective and retrospective liability have one thing in common. In both cases, people might be held liable for the negative effects of their actions even if they were unknown to them and could not reasonably have been foreseen. However, they differ in that with prospective liability people are only held liable for the negative effects of their actions if they were informed beforehand of the burdens they would be required to bear if some accident occurs, whereas in retrospective liability people are held liable for the consequences of their actions even if they were not made aware of the burdens they would be required to bear were their actions to trigger negative effects.

Proponents of the EIO accept the prospective application of strict liability principles but not the retrospective application. That is, they do not take issue with all of the principles that attribute liability for actions whose negative effects could have not been foreseen, such as principles of prospective strict liability. However, they do take issue with the application of principles that make agents bear burdens they could not have expected being required to bear. For this reason, it seems that the concern behind the EIO is about making agents bear burdens they could not have expected being required to bear. This rationale applies to historical emissions because if people did not know and could not reasonably have known about the negative effects of their actions, they could not expect being required to bear the burdens associated with these negative effects.

It is plausible to assume that this concern might be grounded in fairness considerations. Arguably, it is unfair to make people bear burdens they could not have expected being required to bear because this undermines their ability to act as rational planners and executors of plans. Existing laws and regulations, or, in Rawlsian terms, the rules of the basic structure, provide the background for people’s legitimate expectations (Rawls 1971). This stable and relatively permanent framework of expectations provides in turn the basis on which rational planners can consistently and effectively pursue their own ends (Buchanan 1975, p. 422). That is, when people plan and execute their life plans, they do so against a background of legitimate expectations provided by current regulations and law. These include, among others, expectations about what they are permitted to do and what the consequences of their actions would be if they act against current regulations and laws, including what kind of burdens they might be required to bear in such circumstances. It is on this basis that people develop their mid- and long-term plans, which constitute a solid basis for them living a good life (Rawls 1971, pp. 497–516; Williams, in Smart and Williams 1973, pp. 116–117).

However, if people are required to bear unexpected burdens, this frustrates their legitimate expectations and undermines their ability to pursue their mid- and long-term life plans, which in turn has pernicious effects on their ability to live a good life. For instance, bearing unexpected burdens undermines the background of overall resources on which they rely to go about their lives. Hence, imposing unexpected burdens on people harms them in morally significant ways, undermining their ability to pursue their life plans according to their own conception of the good life (Meyer and Sanklecha 2011, 2014).

According to the interpretation I have offered here, the problem that some climate scholars might see with the retroactive application of strict liability with the PPP can be understood to emerge from these fairness considerations. The motivation behind the EIO might be that people should not be required to bear the burdens associated with tackling the negative impacts of climate change because they could not have expected being required to bear these burdens and that imposing these burdens on them would be unfair because it would ultimately undermine their ability to execute their mid- and long-term plans. Thus, the EIO might be grounded in a fairness concern that applies where people did not have the relevant information and could not be expected to have had it, either about the relevant moral facts or about the possibility of being held liable for certain costs.

### Fairness Considerations, the Beneficiary Pays Principle, and Replies to Some Objections

In the previous subsection, I argued that the EIO might be grounded in fairness considerations concerning the harmful impacts of the imposition of unexpected burdens on people that affect their planning and execution of life plans. In this subsection, I propose to assess how this concern may also appear in the application of the BPP.

When individuals pursue certain life plans, they do so against a background of resources that are available to them now and will be in the future. In industrialized countries, this background is heavily influenced by the benefits accumulated over centuries from emission-generating activities (Meyer and Sanklecha 2011, 2014). Industrialized countries and their populations have long relied on the use of these resources. The longer they have been relying on these resources, the stronger the expectations are that they will be able to use them in the future, and the stronger is the importance of the availability of the resources to achieving their plans (Moore 2017). Benefits acquired through emissions-generating activities constitute the background conditions against which rational beings plan and execute their life plans in industrialized countries.

The BPP affirms that beneficiaries should devote these resources to tackling climate change. This means that these benefits will be taken from those who have been relying on them for a long time and that the life plans and expectations depending on the use of these resources will therefore be frustrated. Note that this leaves us with the same problem that we faced with the PPP when applied as a retroactive strict liability principle. In both cases, people are asked to bear burdens they could not have expected being required to bear, which has important moral implications for the development of their mid- and long-term life plans. Thus, the same fairness concern appears again and affects the BPP.

Admittedly, the force of this objection depends on how long beneficiaries have enjoyed these benefits before knowing about their connection to the injustice or undeserved harm, because that will determine how much of their life plans rely on the use of the benefits and the extent to which they are unexpectedly frustrated. With climate change, the benefits that people enjoyed before learning of the harm attached to them are huge. Very likely, most of the basic infrastructure of highly industrialized countries was developed before people learned about the negative effects of climate change. This also explains why the BPP significantly frustrates people’s life plans: because long-lasting benefits have also solidified expectations about long-held mid-term and future plans.

Defenders of the BPP might object that the frustration of people’s legitimate expectations is not as worrisome as the previous argument suggests and even that it is not necessarily unfair to frustrate their expectations. Perhaps these expectations should not be so strongly protected. Hence, the fact that beneficiaries are burdened should not be a reason to refrain from making them bear the costs associated with addressing climate change. The BPP’s defenders might argue that if we share the concern, as we should, that those negatively affected by climate change should not bear the associated burdens, someone else should. In this sense, being burdened is simply the consequence of having to deal with certain justice claims. These burdens might be justified, for instance, because they help in minimizing injustices. This is what Alexandra Couto has called the minimizing injustice argument in support of the BPP (2018, p. 2179). One might even argue analogously that wealthy people will also be burdened when new taxes are imposed on them to tackle unjust distribution in a society. But the burdens created by taxing wealthy people might not trigger fairness concerns when we weigh these burdens against other distributive justice considerations, such as that arising from the regrettable situation of victims of injustice or undeserved harm.

Against this objection, we should note that my argument does not even need to rely on the idea that frustrating people’s legitimate expectations is worrisome, unfair, or even something we should avoid. Instead, remember that my argument is conditional in nature. My point is that *if* the PPP is affected by the EIO and the motivation behind the EIO is based on these fairness considerations, *then* the same fairness considerations apply to the BPP in the context of climate change. Hence, my initial argument still holds: at least some concerns behind the EIO also affect the BPP and to the same extent as the PPP, which undermines moving from the PPP to the BPP based on these grounds.

Admittedly, one might believe that frustrating legitimate expectations is not unfair under these circumstances and that minimizing the suffering of victims should take priority over these considerations. However, in the absence of further explanations, this minimizing injustice argument cannot on its own provide specific support only to the BPP. If what matters is to avoid a situation in which the victim alone bears the burdens, other principles could also be said to achieve this (Couto 2018, p. 2180). These include principles that distribute remedial duties according to whom has the highest capacity or just randomly, and even more interestingly, principles of strict liability such as the PPP itself. The point here is not only that the minimizing injustice argument cannot provide specific support only to the BPP but that such an argument can also provide support to the PPP. Thus, again, such an argument cannot provide a reason to support the BPP that cannot be equally applied to the PPP.

## Conclusion and the Way Forward

Various climate ethicists have proposed moving from the PPP to the BPP as a backward-looking principle of climate justice based on the CO or the EIO. Implicitly, they have assumed that the BPP is not vulnerable to these objections. In this paper, I have challenged that assumption and argued that moving from the PPP to the BPP in response to any of these objections might be unjustified because the BPP may be affected by the same objections or by the same considerations that give rise to these objections.

First, I have shown that the BPP is subject to the CO. In a nutshell, if the principle requires the benefits from emission-generating activities to be used to balance the harm associated with these emissions, in adaptation and compensation cases, we need to determine where the harm has occurred or will foreseeably occur. That is, we need to discriminate between environmental harmful effects that have been caused or will be caused by climate change and those that have not and will not be caused by climate change, but just by natural climate variability. According to the BPP, beneficiaries should bear the burdens associated with the former but not with the later. This requires proving the same level of causation as that involved in the CO.

Second, I have argued that the EIO might be motivated by fairness considerations arising from the frustration of people’s legitimate expectations and the truncation of their mid- and long-term life plans when they are required to bear burdens they could not have expected being required to bear. I have shown that this concern also appears in the case of the BPP because those who would be required to bear the costs of climate change through having benefitted from it have relied on these benefits for a long time. Their life plans and expectations about their future depend on the idea that they will be able to continue to use these resources, and these people will be harmed if the resources needed to address climate change-related problems are taken away from them. Hence, if the EIO is motivated by these fairness considerations, these considerations apply to both the PPP and the BPP. Again, this undermines the move from the PPP to the BPP based on these grounds.

Finally, I have argued that even if we believe that the frustration of legitimate expectations and mid- and long-term life plans is not a cause for very serious concern, this does not affect my main point, which is conditional in nature. The idea is that if one believes that these considerations are relevant, then one should believe that they are relevant for both principles. But if one believes that they are not that relevant, then they are not relevant for either of these principles. Therefore, my main point remains untouched: one cannot justify moving from the PPP to the BPP based on this consideration.

Some might wonder where these conclusions lead. Let me lay out some of the possible implications of my analysis and thus some of the ways forward. First, if both principles face the same problems, then defenders of the BPP might want to reconsider their scepticism towards the strict liability form of the PPP. But, second, if advocates of the BPP insist on defending the purported superiority of this principle, they need to reply to these challenges or bring forward other reasons to move from the PPP to the BPP that might have not been addressed in this paper. In this sense, this paper can be either taken as a light defence of the PPP, showing that the challenges it faces do not uniquely apply to this principle, or as agenda-setting for defenders of the superiority of the BPP.

Third, others might think that if both the PPP and the BPP face these problems, we should discard backward-looking principles altogether and instead adopt fully forward-looking principles. However, in my view, this would be too hasty a conclusion, and it was not the intention of this paper to imply that. Instead, I hope that these results stimulate research on the challenges faced by these principles in grounding a robust climate justice account on backward-looking principles. If these challenges cannot be met, then it might be time to discard backward-looking principles altogether, at least for what concerns historical emissions. However, I believe that hope should prevail here and that more research should be done before reaching such a conclusion.

## Data Availability

Not applicable.

## Footnotes

1 Emphasis mine. For more information: https://www.forbes.com/sites/jeffmcmahon/2015/11/30/obama-says-u-s-accepts-its-responsibility-for-climate-change/?sh=173efb711fe5

2 Arguably, the BPP has been conceived as a hybrid principle that combines both backward- and forward-looking intuitions (see Sect. 3).

3 Other objections include the non-identity problem (Kumar 2003; Duus-Otterström 2014) and the dead-polluters objection (García-Portela 2019; Francis 2020). Whether and to what extent any of these objections constitute reasons to move from the PPP to the BPP is not discussed here.

4 Even though we might not be fully sure that certain emissions did or will cause harm, it might simply be extremely likely that they did or will do so. For a discussion about difference-making of additional emissions, see: (Kagan 2011; Broome 2019).

5 The CO, as it is described here, is related but independent from other causation concerns that might arise in the context of responsibility for climate change-related harm. An additional worry might be that it is so far very difficult to know whether an agent’s emissions have caused or contributed to cause some specific harmful weather events. Unlike in my CO, the scepticism here is not about the connection between emissions generally and local impacts, but rather about *someone’s* emissions and local impacts. Arguably, this can be a challenge for distributing causal and thus moral responsibility to individual agents. However, this worry might be less concerning. Arguably, once one can link specific weather events with anthropogenic climate change, one could adopt a proportional division of responsibility even if proportional causal responsibility cannot be proven (see, for instance, Wündsich 2019; Harrington and Otto 2019). Quite obviously, notice that this objection would not apply to the BPP because this principle does not distribute responsibility according to emissions, but to benefits. For that reason, I do not engage in this discussion here. I thank an anonymous reviewer for suggesting this clarification.

6 If we take 1990 as the cut-off date, historical emissions from 1750 to 1990 represent 48.6% of overall emissions. If we take 1995, historical emissions represent 55.6% of overall emissions. These refer only to CO_2_ emissions. Earlier emissions, until roughly 1960, are rough estimates. Data source: Hannah Ritchie and Max Roser 2017. ‘CO_2_ and Greenhouse Gas Emissions’. Published online at OurWorldInData.org (https://ourworldindata.org/co2-and-other-greenhouse-gas-emissions). Last revision: August 2020.

7 Arguably, the PPP could be understood as a principle of fault liability for later emissions. However, this does not affect the conclusions of this paper. If the EIO does not apply and the PPP works as a principle of fault liability, this also undermines moving from the PPP to the BPP, but I do not discuss this issue further here.

8 Those who believe that engaging in emissions-generating activities cannot be described as an injustice because of the EIO focus on ‘undeserved harms’ (see Meyer and Roser 2010; Meyer 2013; Caney 2006). But others believe that regardless of the EIO these activities could constitute an ‘objective injustice’ (Duus-Ötterstorm 2014) if considered from a time-neutral perspective (Bell 2011; Thompson 2017). The conclusions I present here are valid for both understandings of the principle.

9 The version of the principle I present here combines both the ‘unjust enrichment BPP’ and the ‘wrongful enrichment BPP’ (Page 2012; Heyd 2017), both of which have been discussed in the literature. The first does not require that perpetrators are culpable, whereas the second requires that the actions triggering the original injustice or undeserved harm were committed under culpable conditions. Arguably, a ‘wrongful enrichment BPP’ could be applied in circumstances when emissions-generating activities can no longer be considered innocent. But only the ‘unjust enrichment BPP’ can account for historical emissions. I here propose a formulation that includes both possibilities because this constitutes a broader climate justice principle that covers both historical emissions and later possibly culpable emissions.

10 Emphasis mine.

11 This does not mean that the BPP can also be partly understood as being beneficiary-centred. I argue that for the principle to work as a genuine climate justice principle, it needs to at least also be victim-centred in these ways, for the reasons provided in the main text.

12 Emphasis mine.

13 Two things are worth noting. First, the EIO has been emphasized more than the CO. However, the CO has been mentioned by Gosseries (2004, p. 54), Baatz (2013, pp. 95–97; 2016), Heyd (2017, p. 27), and Walliman-Helmer et al. (2018, pp. 45–46 fn.3) as an objection against the PPP but not against the BPP. Second, the principles of modified strict liability (Caney 2010) and limited liability (Bell 2011) also include this move. These principles involve applying the PPP to identify the duty bearers but limiting the extension of their duties only to the benefits acquired through their emissions (Heyward, 2021). This principle can be read as a version of the BPP that only applies when the beneficiaries are also involved in the harmful action. Here, I understand Caney’s and Bell’s positions as being affected by my argument because they move from the PPP to the BPP when determining the extension of duties.

14 Emphasis mine.

15 Emphasis mine.

16 Admittedly, the objection might be grounded in considerations other than fairness. Here, I only show that there is a plausible interpretation of the EIO based on fairness considerations and that these also apply to the BPP. This is enough to undermine the move from the PPP to the BPP, at least to some extent. If the EIO were grounded in other considerations, they might or might not apply to the BPP. However, to my knowledge no one has provided a deeper explanation of the EIO in other terms, and exploring such an alternative explanation here is not possible due to space constraints. In any case, this should not be a major problem if results are read in a conditional way. My point here is that *if* the EIO is based on fairness considerations of the sort advanced here, then they also apply to the BPP.

17 Other terms, such as strict ‘legal’ and ‘moral’ liability, are frequently used to express the same distinction (Wündisch 2017).

18 Examples of regulatory frameworks of environmental and toxic pollution include the Comprehensive Environmental Response, Compensation, and Liability Act (CERCLA) in the USA and the 2004 Environmental Liability Directive in Europe. For an analysis of how legal systems deal with strict liability for environmental pollution and the possibility of applying these strict liability principles to the case of climate change, see (Farber 2017).
